# Case Report: Successful endoscopic resection of a huge rectal tumor extending to the dentate line and associated with a diverticulum by endoscopic submucosal dissection

**DOI:** 10.3389/fmed.2026.1815880

**Published:** 2026-04-10

**Authors:** Bing-Jie Yan, Yu-Fan Li, Ze Qi, Ming-Wei Zhang, Lei Li

**Affiliations:** 1 Department of Gastroenterology, The Affiliated Hospital of Shandong Second Medical University, Weifang, Shandong, China; 2 School of Clinical Medicine, Shandong Second Medical University, Weifang, Shandong, China

**Keywords:** dentate line, endoscopic submucosal dissection, huge rectal tumor, rectal diverticulum, rectal mucosal carcinoma

## Abstract

**Background:**

Rectal tumors extending to the dentate line (RTDL) represent a distinct subtype of rectal neoplasms due to the unique anatomical features of the dentate line, for which endoscopic submucosal dissection (ESD) has proven to be an effective therapeutic approach. Furthermore, when tumors invade diverticula, the likelihood of perforation during ESD markedly escalates. Reports detailing the simultaneous management of these three high-risk factors-large tumor size, involvement of the dentate line, and association with a diverticulum-are scarce. Thus, this paper outlines the diagnostic and therapeutic process for a patient who underwent successful endoscopic resection of a huge rectal tumor with these combined challenging features by ESD.

**Case summary:**

A 70-year-old male patient was admitted with a history of “altered bowel habits” persisting for 2 years. Subsequent examinations, including a colonoscopy, led to the diagnosis of a lesion with high-grade intraepithelial neoplasia and focal intramucosal carcinoma. The lesion, measuring approximately 7 cm × 8 cm, extended to the dentate line and was accompanied by a diverticulum. Following a multidisciplinary consultation, the patient underwent radical resection through endoscopic submucosal dissection (ESD). The procedure successfully managed the affected diverticulum without complications. Postoperative histological analysis of the en bloc specimen confirmed a conventional serrated adenoma with high-grade intraepithelial neoplasia and focal intramucosal carcinoma (pTis). The resection margins were negative (R0 resection), and there was no lymphovascular invasion. At 1-year postoperative follow-up, no tumor recurrence or distant metastasis was observed, and anal function was preserved.

**Conclusion:**

This case demonstrates the feasibility and curative effect of ESD for large intramucosal rectal carcinoma involving the dentate line and associated with a diverticulum. However, larger cohort studies and long-term follow-up are required to validate the generalizable safety and efficacy of this approach.

## 1 Introduction

Rectal tumors extending to the dentate line (RTDL) represent a distinct subtype of rectal neoplasms. While a consensus on the optimal treatment remains elusive, endoscopic submucosal dissection (ESD) is considered a viable option (1–4). The intricate anatomy of the dentate line significantly complicates traditional ESD techniques due to its dense network of blood vessels and nerves, increasing the chances of bleeding, infection, and postoperative pain. Moreover, the limited anal canal diameter, combined with the constant contraction of the anal sphincter muscle, limits the surgical workspace (5, 6). In addition, when RTDL infiltrates into the diverticulum, the risk of perforation during resection is increased due to the absence of a muscular layer in the diverticulum (7). While endoscopic submucosal dissection (ESD) for rectal tumors crossing the dentate line or those associated with diverticula has been individually reported, cases combining a large tumor size with both anatomical challenges are exceptionally rare. This study elucidates the diagnostic and therapeutic approach for a patient who successfully underwent ESD for a huge rectal lesion that was not only associated with a diverticulum but also extended to the dentate line.

## 2 Case description

A 70-year-old man presented to our hospital with a 2-year history of altered bowel habits. He had been experiencing yellow, loose stools with occasional blood, occurring 2–3 times daily, without other significant symptoms. The patient had taken antidiarrheal medications, with poor therapeutic efficacy. He had no underlying medical conditions, low work pressure, and no history of smoking or alcohol consumption. The patient denied any family history of hereditary diseases. No positive physical signs were found upon admission, and his vital signs were as follows: Body temperature 36.2, heart rate 73 beats/min, respiratory rate 18 breaths/min, blood pressure 144/92 mmHg, body mass index (BMI) 27.7 kg/m^2^. Upon admission, the blood routine, urine routine, fecal routine, biochemistry tests, immune indexes and infection indexes were all within normal ranges. Abdominal CT scan identified localized rectal wall thickening. Colonoscopy revealed a mass encompassing two-thirds of the rectal circumference, extending to the dentate line, with localized protuberance and central erosion (7 cm × 8 cm, Paris classification 0-IIa + IIc, irregular surface pattern). Under narrow-band imaging (NBI), the lesion presented as a nodular, cerebriform lateral spreading tumor (LST) with well-defined margins. It showed increased, disorganized microvascularity and irregular pit patterns (type IV/V), and focal mild erosion was noted. Five biopsy specimens were collected for pathological examination (Supplementary Figure 1). Subsequent pathology results indicated high-grade intraepithelial neoplasia of the rectal glandular epithelium, with focal intramucosal carcinoma. The presence of the muscularis mucosae was inconclusive, raising concerns about deep invasion. To further assess the depth of invasion and rule out lymph node metastasis, an additional pelvic MRI was conducted, which confirmed the absence of distant or lymph node metastasis. The pelvic MRI examination revealed an irregular soft tissue mass measuring approximately 2.6 cm × 2.1 cm protruding into the lumen on the left wall of the mid rectum, with hyperintense signals on DWIBS. The mass had ill-defined borders, and the serosal surface of the rectum involved by the lesion remained intact. In addition, no distant tumor invasion or lymph node metastasis was identified on MRI. The discrepancy between the endoscopic measurement (7 cm × 8 cm) and the MRI measurement (2.6 cm × 2.1 cm) is attributable to the fact that colonoscopy captures the entire lateral spreading component of the tumor, whereas MRI primarily visualizes the invasive portion with associated wall thickening. The lesion exhibited extensive superficial spreading beyond the central invasive focus, which is a typical feature of lateral spreading tumors.

We determined that the lesion was an early rectal carcinoma, likely confined to the mucosal layer. After multidisciplinary discussion (MDT) involving gastroenterologists, gastrointestinal surgeons, radiologists, and pathologists, we carefully weighed the risks and benefits of surgical versus endoscopic management. Given the patient’s advanced age (70 years) and the tumor’s extension to the dentate line, where surgical resection carries a substantial risk of anal sphincter damage and potential impairment of postoperative continence. Although the preoperative assessment could not definitively exclude deep submucosal invasion, the absence of lymph node metastasis on MRI, the endoscopic features (Paris 0-IIa + IIc with superficial elevation), and the patient’s strong desire for sphincter preservation collectively supported an initial attempt at curative endoscopic resection. The MDT consensus was that ESD offered the best balance of oncologic safety and functional preservation for this high-risk lesion, with the clear understanding that further surgical resection would be pursued promptly if postoperative pathology revealed deep invasion beyond the appropriate indications for endoscopic treatment.

The case presented three challenges. First, the huge tumor volume surrounding a large portion of the lumen made resection difficult due to limited visibility, increasing the chances of residual disease. Secondly, the tumor’s involvement with the dentate line, rich in blood vessels and nerves, raised the risk of postoperative bleeding and pain. Lastly, the tumor, combined with a muscularly unsupported diverticulum, significantly increased the risk of perforation during surgery.

The ESD procedure is shown in Supplementary Figure 2.

(1) Submucosal injection: To maintain a safe cushion and ensure optimal surgical exposure, a submucosal injection solution was injected into the submucosal layer around the lesion to lift the lesion from the muscularis propria. Epinephrine was added to the submucosal injection solution to prevent intraoperative and postoperative bleeding.
(2) Marking: Given the clear intestinal visual field and well-defined tumor margins, routine electrocoagulation marking was not performed at the tumor margins.
(3) Incising the tumor: To address the large circumferential lesion and narrow anal canal, a strategy of “retrograde dissection from the anal side” was employed. The procedure began by incising the anal side of the lesion, allowing the endoscope to be reversed for a better approach. The submucosal dissection was then carried out in a stepwise, proximal direction (from the anal verge upwards). Besides, to minimize intraoperative bleeding, we adopted the meticulous prophylactic electrocoagulation approach for all visible perforating vessels larger than 1 mm.
(4) Diverticulum sealing: As a critical preventive measure against perforation, the orifice of the diverticulum was sealed with three metal clips. This provided a clear plane of dissection and mitigated the risk of postoperative diverticulitis and perforation.
(5) Wound management: Considering the risk of stenosis in the narrow anal canal, the post-ESD mucosal defect was intentionally left open. Primary closure with clips was avoided to prevent potential clip displacement and postoperative stricture.

Given the patient’s advanced age, standard fasting was avoided to prevent cardiac insufficiency. Instead, the patient was allowed to consume water and oral nutritional supplements starting on the second day after the procedure, to ensure adequate caloric intake while minimizing fecal formation.

The patient resumed a liquid diet on the third day after surgery without encountering any complications such as bleeding, anal pain, bacteremia, or intestinal stenosis. By the seventh postoperative day, the patient had progressed to a semi-liquid diet and was discharged from the hospital. The en bloc resected specimen was sent for histopathological evaluation. According to the World Health Organization (WHO) Classification of Tumors of the Digestive System (6th edition) (8), the final diagnosis was a conventional serrated adenoma with high-grade intraepithelial neoplasia and focal intramucosal carcinoma (pTis). The conventional serrated adenoma is a recognized serrated polyp subtype characterized by distinctive serrated architecture and variable degrees of dysplasia. In this case, the high-grade dysplastic component progressed to intramucosal carcinoma, which remains confined to the lamina propria without invasion beyond the muscularis mucosae. This corresponds to Vienna classification category 4 (non-invasive high-grade neoplasia). Importantly, the horizontal and vertical resection margins were negative (R0 resection), and there was no evidence of lymphovascular invasion. At 1-year postoperative follow-up, no tumor recurrence or distant metastasis was observed, and anal function was preserved. The timeline with relevant data from the episode of care is shown in Table 1.

**Table 1:** The timeline with relevant data from the episode of care.

Timeline	Admission day 1	Admission day 2	Admission day 3	Admission day 4	2 days after ESD	7 days after ESD	1 year after ESD
Clinical date	The blood routine, urine routine, fecal routine, biochemistry tests, immune indexes and infection indexes were all within normal ranges. Abdominal CT scan identified localized rectal wall thickening.	Colonoscopy revealed a mass encompassing two-thirds of the rectal circumference, extending to the dentate line, with localized protuberance and central erosion.	Pathology results indicated high-grade intraepithelial neoplasia of the rectal glandular epithelium, with focal intramucosal carcinoma. Abdominal MRI confirmed the absence of distant tumor metastasis.	The patient underwent ESD surgery successfully without complications.	The patient was allowed to consume water and oral nutrition.	The postoperative pathology revealed a conventional serrated adenoma with high-grade intraepithelial neoplasia and focal malignant transformation (intramucosal carcinoma), confirming curative resection. The patient was discharged from the hospital.	The endoscopic follow-up at the1 year after ESD showed no residual or recurrent lesions.

## 3 Discussion

Before the advent of endoscopic methods, rectal tumors that affect the dentate line (RTDL) were mainly treated through surgical excision. However, surgery in this region carries a potential risk of sphincter damage, which may compromise anal function and diminish postoperative quality of life. Compared with conventional surgical approaches, Endoscopic submucosal dissection (ESD) presents distinct benefits, including preservation of anal function, reduced trauma, faster postoperative recovery, and decreased patient discomfort (9–11). The comparison table of different treatment methods for RTDL is shown in Table 2.

**Table 2:** The comparison table of different treatment methods for RTDL.

Comparison dimension	Endoscopic submucosal dissection (ESD)	Laparoscopic radical resection	Transanal endoscopic microsurgery (TEM)
Indications	Lesions confined to the mucosal layer or superficial submucosal layer, with the need to preserve anal function	Lesions invading the deep submucosal layer or beyond, with risk of lymph node metastasis; tumors with a diameter of >5 cm and extensive invasion	Low rectal lesions (distance from anal verge <5 cm), diameter 2–5 cm, no extensive invasion or lymph node metastasis
Trauma degree	Minimally invasive (no abdominal incisions, transanal operation)	Moderate (3–4 small abdominal incisions, 0.5–1.0 cm in length)	Minimally invasive (transanal operation, no abdominal incisions)
Impact on anal function	Low (precise dissection of lesions, minimal damage to anal sphincter)	Moderate-high (possible sphincter damage, some patients require enterostomy)	Moderate-low (resection under direct vision, controllable risk of sphincter damage)
En bloc resection rate (cases involving dentate line)	85.7–100%	95–100%	90–95%
R0 resection rate (cases involving dentate line)	53.3–93.8%	85–95%	88–92%
Operation duration	60–120 min	120-180 min	90-150 min
Postoperative recovery period	7–10 days	10-14 days	8-12 days
Recurrence rate	4.5–7.3% (1–2 years postoperatively)	3–5%(3 years postoperatively)	5–8% (2 years postoperatively)
Complication risk	Bleeding (3–5%), perforation (2–4%), mild postoperative pain	Infection (5–8%), intestinal obstruction (3–6%), stoma-related complications (10–15%)	Bleeding (4–6%), rectal perforation (2–3%), anal stenosis (3–5%)
Core advantages	Preserves organ integrity, high quality of life; minimally invasive with fast recovery; applicable to complex cases such as those with diverticula.	Strong radical effect, thorough lymph node dissection; applicable to invasive lesions	Clear surgical field, precise resection; good adaptability to low-position lesions
Limitations	great operational difficulty, high technical requirements for endoscopists; not applicable to deeply invasive lesions or cases with lymph node metastasis	Relatively large trauma, slow postoperative recovery; some patients lose anal function	Narrow applicable lesion range (diameter <5 cm); long learning curve

In comparison to the proximal rectum and colon, the unique anatomical and physiological features of the dentate line impose specific challenges for ESD. First, the squamous epithelium of the anal canal contains a dense rectal venous plexus and abundant sensory nerves, increasing the risk of intraoperative bleeding and postoperative pain. Second, the rectal venous plexus drains directly into the systemic circulation, bypassing the portal system, which elevates the risk of bacteremia. Third, the dentate line represents a transitional zone where the luminal diameter narrows to only 2.5–3 cm, compared with 4.0–5.0 cm in the upper rectum. This narrowing is further accentuated by the surrounding internal and external anal sphincters, which restrict both surgical maneuverability and visualization (12, 13). Collectively, these distinctive features contribute to the technical difficulty of performing ESD at this site and explain the relative scarcity of research focused on this specific anatomical region.

Despite these challenges, accumulating evidence supports the feasibility of ESD for RTDL. International reports have shown that ESD achieves en bloc resection rates ranging from 85.7 to 100%, and R0 resection rates ranging from 53.3 to 93.8% for RTDL, with recurrence rates of 4.5% at 4–6 months and 7.3% at 10 months postoperatively (14–16). Similar findings have been reported in domestic studies. Li et al. reported an en bloc resection rate of 87.5% and a negative margin rate of 87.5% for RTDL treated by ESD, with no recurrence observed during a follow-up period of 12–48 months (17). Another study demonstrated successful en bloc resection of 45 laterally spreading tumors involving the dentate line in a single ESD session, with no recurrence or residual disease after a mean follow-up of 30.2 months (range: 10–46 months) (18). These findings confirm the safety and efficacy of ESD for treating RTDL.

When a rectal tumor is associated with a diverticulum, the absence of a muscular layer in the diverticular wall markedly increases the risk of perforation during endoscopic resection (18). Nevertheless, ESD has been shown to be a viable option in this context. A Japanese study of 12 diverticulum-associated colorectal tumors treated by ESD reported a 100% R0 resection rate with no intraoperative complications or residual disease (19). Similarly, a retrospective study demonstrated R0 resection rates of 95–96.3% and curative resection rates of 88.9–90% for diverticulum-associated colorectal tumors treated by ESD, with only one case of postoperative bleeding that was successfully managed endoscopically (20, 21). These data support the role of ESD as an effective therapeutic strategy for diverticulum-associated colorectal tumors.

Our case extends the existing literature by demonstrating the successful application of ESD in a complex scenario where dentate line involvement and a diverticulum converge in a large lesion. The curative outcome, confirmed by R0 resection and the absence of lymphovascular invasion, illustrates the feasibility of this approach even under challenging anatomical conditions in this patient. Several intraoperative technical adaptations were critical to achieving this outcome. First, to address the large circumferential tumor in the narrow anal canal, we employed retrograde dissection from the anal side, initiating the incision at the dentate line using a retroflexed endoscopic view and proceeding proximally in a stepwise manner. This approach optimized visualization and maintained a controlled dissection plane. Second, to minimize bleeding risk, we added epinephrine to the submucosal injection solution and applied a prophylactic electrocoagulation strategy to all visible submucosal vessels, adhering to a “coagulate-before-cutting” principle. Third, we closed the diverticulum orifice with metal clips to prevent postoperative diverticulitis and perforation. Finally, we intentionally left the post-ESD mucosal defect unclosed, avoiding primary clip closure that might have led to clip displacement or stricture formation. Notably, the patient resumed oral nutritional supplements on the second postoperative day and a liquid diet on the third day, without experiencing bleeding, significant pain, bacteremia, or stenosis. This uneventful recovery is consistent with the feasibility of this approach in this patient, though further experience is needed to establish generalizability.

However, this report is subject to the inherent limitations of a single case study, which precludes generalization of our findings. The follow-up period of 1 year, while showing no recurrence, is relatively short, particularly for a large lesion with serrated histology, which may carry a distinct risk profile. Longer-term surveillance is essential to definitively confirm the curative outcome. Additionally, the technical strategies described reflect the experience of a single center and may be operator-dependent; further studies are needed to validate their reproducibility.

## 4 Conclusion

This case demonstrates the feasibility and curative effect of ESD for large intramucosal rectal carcinoma involving the dentate line and associated with a diverticulum. However, larger cohort studies and long-term follow-up are required to validate the generalizable safety and efficacy of this approach.

### 4.1 Patient perspective

The patient reported satisfaction with the treatment outcome. He experienced minimal postoperative pain and was able to resume normal activities within 2 weeks. At the 1-year follow-up, he expressed relief at the absence of recurrence and the preservation of normal anal function, which significantly improved his quality of life.

## Data Availability

The raw data supporting the conclusions of this article will be made available by the authors, without undue reservation.
