# Supplementary material for: Case Report: Successful endoscopic resection of a huge rectal tumor extending to the dentate line and associated with a diverticulum by endoscopic submucosal dissection
Source: Front Med (Lausanne). 2026 Apr 10;13:1815880. doi: 10.3389/fmed.2026.1815880 (PMC13105892; doi:10.3389/fmed.2026.1815880)
Supplement: Supplementary file 1 [file Supplementary_file_1.docx]

Supplementary Material

# Supplementary Figures


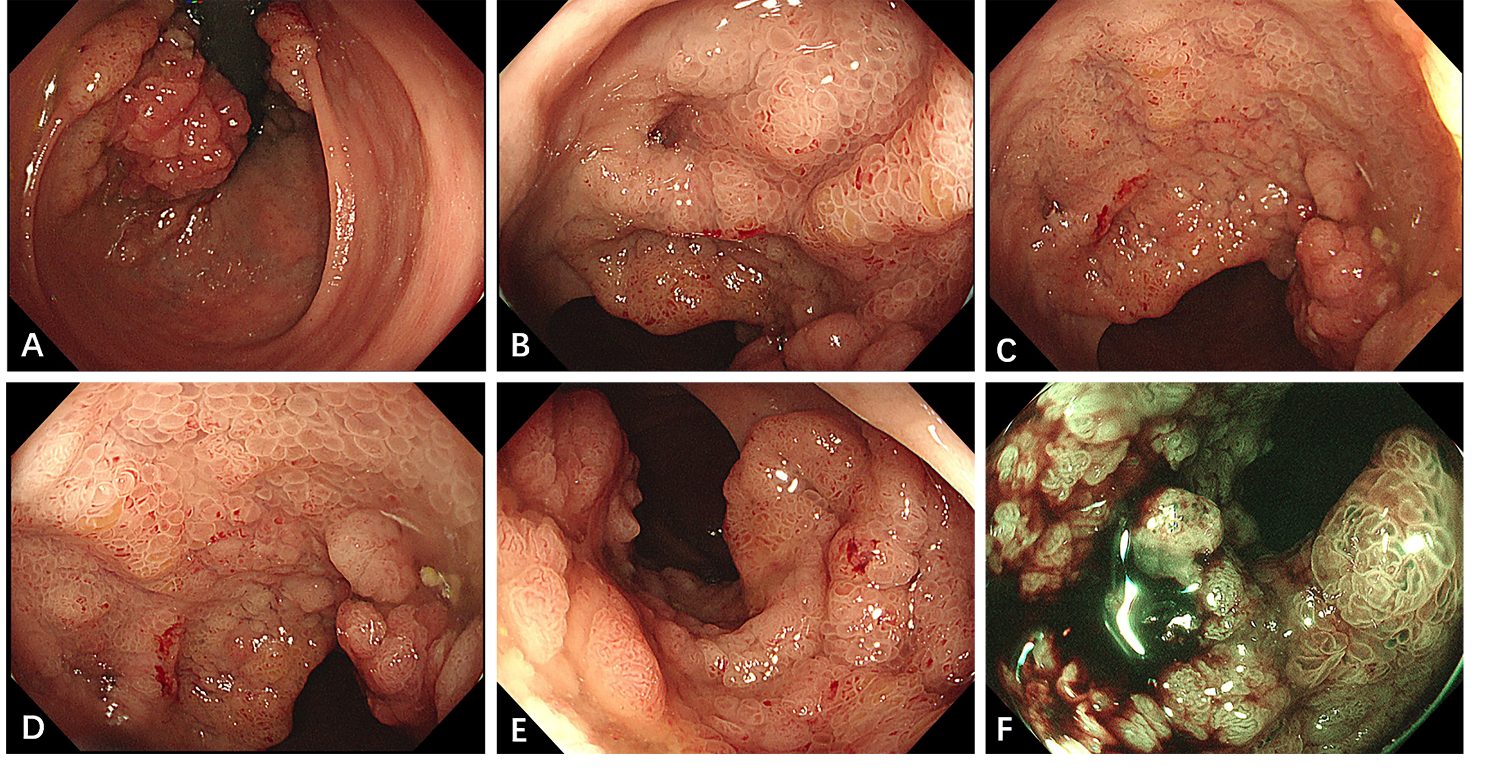


**Supplementary Figure 1.** Colonoscopy Images Related to Lesions: **A-E:** White-light colonoscopy reveals a large, 2/3 circumferential lateral spreading tumor in the mid-to-lower rectum, extending to the dentate line. The lesion exhibits a nodular, cerebriform surface with multiple fused nodules of varying sizes, accompanied by localized protuberance and central superficial erosion. The tumor margin is well-demarcated from the adjacent normal rectal mucosa; **F:** The NBI view demonstrates increased microvascular density, tortuous and disorganized vessels, and irregular pit patterns (type IV/V).


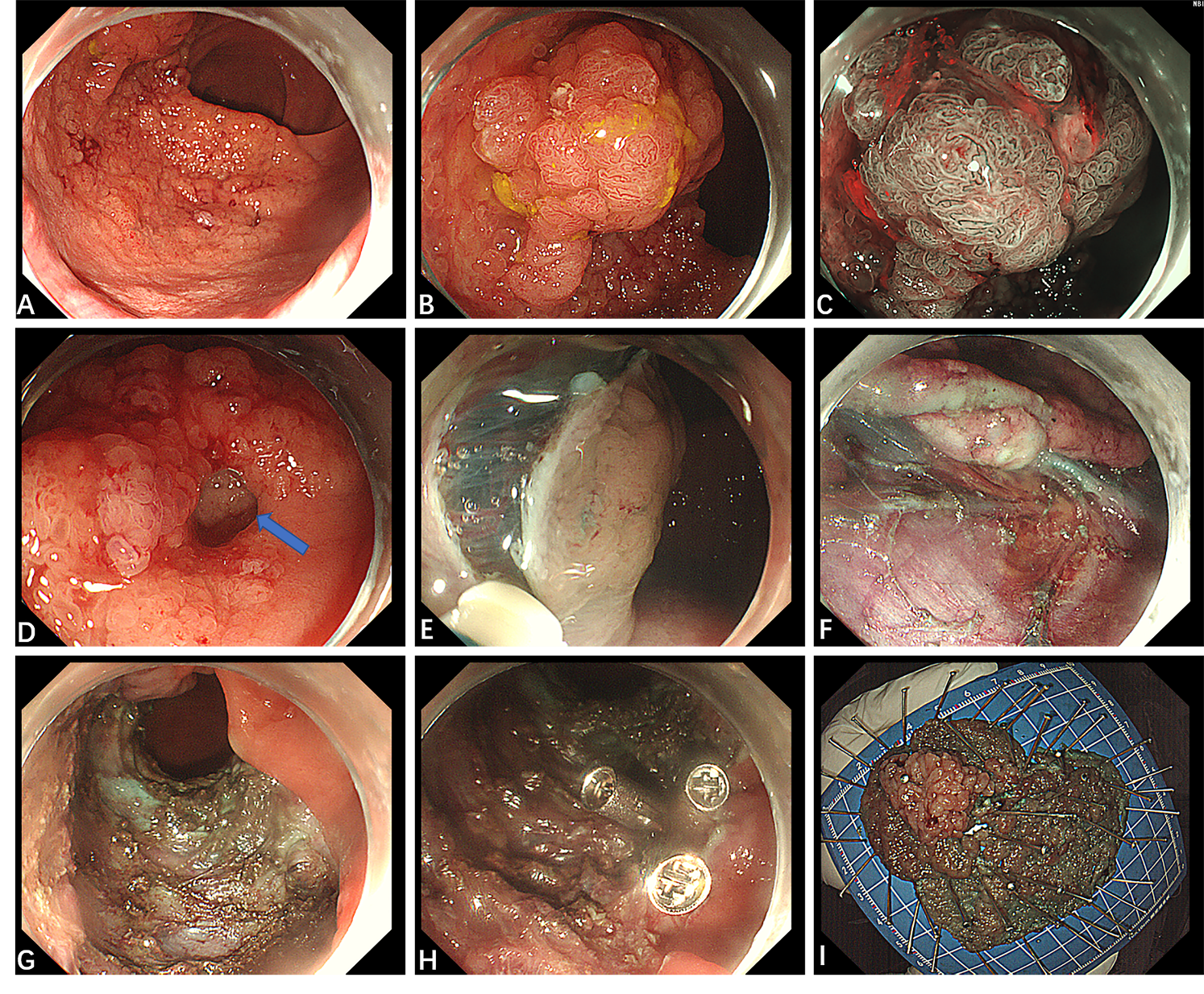


**Supplementary** **Figure 2** ESD procedure images: **AB:** White-light colonoscopy reveals a large, irregular 7 cm × 8 cm mass occupying two-thirds of the rectal lumen and extending to the dentate line; **C:** NBI mode highlights the lesion’s disorganized microvascularity and irregular pit patterns; **D:** A diverticulum (arrow) is visualized on the right side of the lesion, with the tumor involving its entire orifice; **E:** Submucosal lifting is performed, followed by circumferential incision into the superficial submucosa using a Dual knife; **F:** Perforating vessels are prophylactically electrocoagulated with hot biopsy forceps to prevent bleeding; **G:** The post-resection mucosal defect is shown after complete lesion dissection; **H:** The diverticulum orifice is securely sealed with three metal clips to avoid perforation; I: The en bloc resected specimen measures 7.5 cm × 5.5 cm × 1.3 cm, pinned for pathological evaluation.
